# The ameliorating effect of *limosilactobacillus fermentum* and its supernatant postbiotic on cisplatin-induced chronic kidney disease in an animal model

**DOI:** 10.1186/s12906-023-04068-8

**Published:** 2023-07-17

**Authors:** Ahmad Gholami, Nima Montazeri-Najafabady, Yousef Ashoori, Kimia Kazemi, Reza Heidari, Navid Omidifar, Iman Karimzadeh, Mohammad Mehdi Ommati, Seyedeh Narjes Abootalebi, Nasim Golkar

**Affiliations:** 1 Biotechnology Research Center, Shiraz University of Medical Sciences, Shiraz, Iran; 2 Endocrinology and Metabolism Research Center, Shiraz University of Medical Sciences, Shiraz, Iran; 3 Department of Clinical Pharmacy, School of Pharmacy, Mazandaran University of Medical Sciences, Sari, Iran; 4 Pharmaceutical Sciences Research Center, Shiraz University of Medical Sciences, P.O. Box 71348-14336, Shiraz, Iran; 5 Department of Pathology, School of Medicine, Shiraz University of Medical Sciences, Shiraz, Iran; 6 Department of Clinical Pharmacy, School of Pharmacy, Shiraz University of Medical Sciences, Shiraz, Iran; 7 Henan Key Laboratory of Environmental and Animal Product Safety, College of Animal Science and Technology, Henan University of Science and Technology, Luoyang, Henan, 471000 China; 8 Division of Intensive Care Unit, Department of Pediatrics, School of Medicine, Shiraz University of Medical Sciences, Shiraz, Iran; 9 Department of Pharmaceutics, School of Pharmacy, Shiraz University of Medical Sciences, Shiraz, Iran

**Keywords:** Probiotic, Postbiotic, *Limosilactobacillus fermentum*, Renoprotection, CKD mice model, In-vivo study, Integrative medicine

## Abstract

**Background:**

Chronic kidney disease (CKD) is a worldwide public health problem affecting millions of people. Probiotics and postbiotics are associated with valuable compounds with antibacterial, anti-inflammatory, and immunomodulatory effects, preserving renal function in CKD patients. The current study is aimed to evaluate the efficacy of *Limosilactobacillus fermentum* (*L. fermentum*) and its postbiotic in an animal model of cisplatin-induced CKD.

**Methods:**

The animals were divided into four experimental groups (normal mice, CKD mice with no treatment, CKD mice with probiotic treatment, and CKD mice with postbiotic treatment). CKD mice were induced by a single dose of cisplatin 10 mg/kg, intraperitoneally. For 28 days, the cultured probiotic bacteria and its supernatant (postbiotic) were delivered freshly to the related groups through their daily water. Then, blood urea nitrogen (BUN) and creatinine (Cr) of plasma samples as well as glutathione (GSH), lipid peroxidation, reactive oxygen species, and total antioxidant capacity of kidneys were assessed in the experimental mice groups. In addition, histopathological studies were performed on the kidneys.

**Results:**

Application of *L. fermentum* probiotic, and especially postbiotics, significantly decreased BUN and Cr (P < 0.0001) as well as ROS formation and lipid peroxidation levels (P < 0.0001) along with increased total antioxidant capacity and GSH levels (P < 0.001). The histopathologic images also confirmed their renal protection effect. Interestingly, the postbiotic displayed more effectiveness than the probiotic in some assays. The improvement effect on renal function in the current model is mainly mediated by oxidative stress markers in the renal tissue.

**Conclusions:**

In conclusion, it was found that the administration of *L. fermentum* probiotic, and particularly its postbiotic in cisplatin-induced CKD mice, showed promising effects and could successfully improve renal function in the animal model of CKD. Therefore, probiotics and postbiotics are considered as probably promising alternative supplements to be used for CKD.

## Introduction

Chronic kidney disease (CKD) is characterized by irreversible and progressive alteration in the function and structure of the kidney during months or years [1, 2]. It is considered one of the fastest-growing causes of death and is estimated to become the fifth global cause by 2040 [3]. The progression of the disease might be influenced by several factors, such as dietary intake, mental stress, and medications [4]. Current approaches for CKD management include low protein and sodium intake, blood pressure control, and glycemic control [5]. However, no effective therapy exists for this health issue, and innovative strategies are necessary to manage, control, and even treat the disease [5, 6].

*Limosilactobacillus fermentum* (*L. fermentum*) is one of the common probiotic strains in nature [7, 8], usually isolated from fermenting plant material, bread, dairy products, naturally fermented sausages, saliva, and breast milk [8, 9]. Apart from extensive applications in the food industry, there have been many studies regarding the effectiveness of *L. fermentum* [8, 10, 11], for example, prevention and treatment of gastrointestinal diseases, prevention of alcoholic liver disorder, alleviating colorectal cancer risk and a lot more [12, 13], which are summarized in Table 1.

**Table 1:** Various in vivo studies regarding the effectiveness of *L. fermentum*

Study	Finding
The effect of *L. fermentum* in animal models of ethanol-induced liver disease	Considerable decrease in ethanol-induced liver tissue damage [14, 15].
The effect of *L. fermentum* on hypercholesterolemia	Amelioration of hypercholesterolemia by the probiotic’s antioxidant effect, anti-inflammatory effect, and gut barrier function [16, 17].
The effect of *L. fermentum* on colitis	Effective reduction of the symptoms of colitis in mice through different mechanisms, such as modulating the nuclear factor-κB (NF-κB) signaling pathway and ameliorating the inflammation and/or antioxidant properties [18–20].
The effect of *L. fermentum* on sleep disturbance	Efficient amelioration of sleep disturbance produced by the first night effect (FNE) and promotion of non-rapid eye movement (NREM) sleep in mice [21].
The effect of *L. fermentum* on *Helicobacter. Pylori*	Inhibition of the *Helicobacter pylori* colonization [22].
The effect of *L. fermentum* following local administration on vaginal infection	The antimicrobial preventative as well as curative effects against *Escherichia coli* [23].
The effect of *L. fermentum* on colorectal cancer	Attenuation of the risk of colorectal cancer [24, 25].
The effect of *L. fermentum* on aging	Potential decrease of aging symptoms in rats and mice via its various properties, such as antioxidant effects [26, 27].
The effect of *L. fermentum* on renal damage in a systemic lupus erythematosus mouse model	Prevention of the impairment of kidney function and damage through various mechanisms such as reducing blood lipopolysaccharides, reduction of inflammation and oxidative stress, as well as immune complex deposition [28].

The International Scientific Association of Probiotics and Prebiotics (ISAPP) defines postbiotics as the preparation of inanimate microorganisms and/or their components, which induces a health benefit on the host [29]. Different components are considered postbiotics, such as cell-free supernatant, functional proteins, extracellular polysaccharides (EPS), enzymes, cell wall fragments, short-chain fatty acid, and bacterial lysate [30]. Since postbiotics are free of living microorganisms, the possible risks associated with postbiotic use might be fewer than the probiotics while maintaining their effectiveness [30, 31]. Postbiotics could be an attractive alternative for other biotic members. Postbiotics can be absorbed and appropriately metabolized and have shown higher stability, facile transportation, and essential signaling potential with different organs and tissues [32–34].

Furthermore, postbiotics have other favorable properties, including anti-inflammatory, immuno-modulatory, antioxidant, antitumor, anti-hypertensive, infection prevention, anti-atherosclerotic, autophagy, and antiproliferative properties [35–37]. However, there have been fewer investigations that studied the efficacy of postbiotics. To the best of our knowledge, no study has been conducted about postbiotics in kidney diseases.

The present study is aimed to examine the potential efficacy of *L. fermentum* and its postbiotic in cisplatin‑induced CKD in an animal model. Therefore, we assessed various factors, including blood urea nitrogen (BUN) and plasma creatinine (Cr) of serum samples, glutathione (GSH), lipid peroxidation, reactive oxygen species (ROS), and total antioxidant capacity of kidneys. Also, we examined the histopathological consequences of the probiotic and its bioactive metabolites to investigate their efficacy.

## Materials and methods

### Materials

De Man, rogosa & sharpe (MRS) broth medium was prepared from Himedia (India). Cisplatin was obtained from Ebewe Pharma (Austria). Sodium thiopental, tris, potassium chloride (KCl), dichlorofluorescein (DCF), phosphoric acid, thiobarbituric acid, n-butanol, trichloroacetic acid (TCA), ethylenediaminetetraacetic acid (EDTA), and dithio-bis-(2-nitrobenzoic acid) (DTNB, Ellman’s reagent) were purchased from Merck (Germany). Acetic acid, sodium acetate, ferric chloride dihydrate, and 2,4,6-tris(2-pyridyl)-s-triazine (TPTZ) were also from Merck (Germany). All other solvents and reagents utilized to prepare various buffer solutions were of analytical grade and purchased from Merck (Germany). All the preparations were made using deionized water (Direct Q UV3, Millipore, USA).

### Probiotic and postbiotic preparation

*Limosilactobacillus fermentum* PTCC No. 1744 as the probiotic strain was purchased from Persian Type Culture Collection in Iranian Research Organization for Science and Technology (IROST). The bacteria were cultured in MRS broth medium for 48 h at 37 °C under microaerophilic conditions until the stationary phase was achieved. The pH of 7.2 was adjusted for the media. Afterward, the number of viable bacteria was counted by plate counts using MRS agar, and an inoculum of bacteria containing an approximate density of 10^9^ CFU/ml was prepared.

Preparation of postbiotic was done according to the method previously described by Montazeri-Najafabady et al. [38]. Briefly, the bacteria were centrifuged at 4000 *g* and 4 °C for 20 min using a refrigerated centrifuge (Eppendorf 5804 R Germany), and the biomass (including probiotic cells) was collected. The supernatant postbiotic was filtered through a 0.2 μm membrane filter to remove any remaining probiotic bacteria. The filtered supernatant (containing postbiotic) was then lyophilized by a freeze-drier (Alpha 1-2LD Plus, Martin Christ, Germany) and kept at − 20 °C until further use. The final product was held for a maximum of 12 days.

### Experimental design

Twenty male BALB/c mice with an average weight of 22.5 ± 2.5 g and an age of 8 weeks were supplied from the comparative and experimental medicine center of Shiraz University of Medical Sciences. All the animals were housed in standard cages where water and standard food were easily accessible. The mice were kept in suitable conditions under the average temperature of 22 ± 2 °C and humidity of 44 ± 5% with a 12-h light-dark cycle. To inhibit any stress effects on animals, they were allowed to adapt with the new situation for two weeks [39, 40]. All animal procedures were performed under the supervision of the institutional ethics committee of Shiraz University of Medical Sciences, Shiraz, Iran (Ethics committee code: IR.SUMS.REC.1400.057). The ARRIVE guidelines for the care and use of laboratory animals were also followed.

The mice were divided into four experimental groups: normal mice received distilled water (no treatment, Group 1), CKD mice received distilled water (no treatment, Group 2), CKD mice received *L. fermentum* probiotic bacteria (Group 3), and CKD mice received postbiotic (Group 4), each group contained five mice. To induce CKD in the animals, the mice received a single dose of cisplatin (10 mg/kg) through intraperitoneal (i.p.) injection and studied five days later [41]. The probiotic (for group 3) and postbiotic (for group 4) were freshly prepared every day and added to the animals’ water at a proportion of 1:4 probiotic/postbiotic: daily water (15 ml daily water for each mouse). The experiment was performed for 28 days. Sample collections (serum and kidney pieces) were collected for biochemical/histological assessment following deep anesthesia/scarification on day 28.

### Biochemical assay

#### Blood urea nitrogen (BUN) and creatinine (cr) assay

The mice were anesthetized by intra-peritoneal sodium thiopental at 70 mg/kg, and blood samples (5 ml for each piece) were collected carefully from the abdominal vein. Then, the blood samples were clotted at room temperature and centrifuged at 1000 rpm for 25 min to separate serum parts. The obtained serum was kept at -20 °C for further experiments. The blood serum was utilized to assess the experimental animals’ BUN and creatinine as biomarkers of renal injury using an automated biochemistry analyzer (BM/Hitachi 747, Tokyo, Japan).

### Renal oxidative stress (ROS) assay

#### ROS formation

250 mg of the kidney tissue for each mouse was weighed and poured into 2.5 ml of cooled tris buffer (40 mM, pH = 7.4). Then, the tissue was homogenized with a homogenizer (IKA T 25 digital ULTRA-TURRAX^®^). 100 µl of the homogeneous mixture was added to 1ml of cooled tris buffer (40 mM, pH = 7.4), and then 5 µl of DCF solution (1 µM) was added to the obtained mixture. After 30 min incubation at 37 ° C, the fluorescence intensity of the samples was measured at the excitation and emission wavelengths of 485 and 525 nm, respectively, using a fluorimeter (FLUOstar Omega^®^ multifunctional microplate reader, BMG LABTECH, Germany) [42].

#### Tissue lipid peroxidation

The kidney tissue (500 mg) was mixed with 5 ml of KCl solution (1.15% w: v) and homogenized at the temperature of 4 °C. Then, 0.5 ml of the homogenized tissue was blended with 3 mL of 1% m-phosphoric acid and 1 ml of 0.6% w: v of thiobarbituric acid and mixed gently for 5 min. Next, the mixture was heated at 100 °C for 45 min in a water bath. After that, 2 mL of n-butanol was added to the cooled mixture and vortexed for 5 min. After centrifuging the samples, 100 µL of the upper phase (n-butanol phase) was added to a 96-well plate. The absorbance was measured by a spectrophotometer (EPOCH^®^ plate reader, BioTek^®^, USA) at the wavelength of 532 nm [42].

#### Total antioxidant capacity

Ferric reducing antioxidant power or ferric-reducing ability of plasma (FRAP) assay was used to assess the total antioxidant capacity, which is based on the reduction of the complex of ferric tripyridyltriazine (Fe^3+^-TPTZ) to ferrous tripyridyltriazine (Fe^2+^-TPTZ) via the antioxidants of a sample at low pH [42]. The end product, Fe^2+^-TPTZ, shows a blue color in which absorption is measured [43]. Accordingly, 500 mg of the kidney tissue was weighed and poured into 5 ml of cooled KCl solution (1.15% W/V). The tissue was homogenized using the homogenizer. After that, 100 µL of the homogeneous mixture was mixed with 3 mL of FRAP reagent (2.5 ml acetate buffer (300 mM, pH = 3) and 0.25 ml ferric chloride dihydrate (20 mM)) and 0.25 mL of TPTZ solution. Following incubation for 4 min, the mixture was centrifuged at 10,000 rpm for 1 min. Finally, the absorption was read using a cuvette spectrophotometer (BioTEK® Instruments, Highland Park, USA) at the wavelength of 593 nm.

#### Glutathione (GSH) level

The kidney tissue (500 mg) was homogenized in 5 mL of 40 mM EDTA solution at 4 °C to measure the GSH level. Then, 5 ml of the homogenized mixture was mixed with 4 ml of distilled water and 1 ml of TCA (50% W/V) and stirred vigorously. The resulting mixture was centrifuged at 3000 *g* and 4 °C for 15 min. 2 ml of the supernatant was added to 4 ml of 0.4 M Tris buffer and 0.1 ml of 10 mM DTNB (Ellman’s reagent) followed by shaking well. Finally, the absorbance of each sample was measured using the spectrophotometer at the wavelength of 412 nm in less than 5 min [42].

### Histopathological study

As previously mentioned, the kidney samples were preserved in a 10% formalin solution for histopathological assay [44]. The paraffin-embedded kidney tissue sections were prepared and stained with the hematoxylin-eosin dye. The destruction in kidney sections (damage %) was reported by investigating glomerular atrophy, tubular change, interstitial nephritis, and vascular change [44]. A semi-quantitative grading method was applied. The degree of nephropathy changes was calculated compared to the control group.

### Statistical analysis

Statistical analysis was performed using GraphPad software version 8 (v8.4.0, GraphPad Software Inc., San Diego, CA). Comparisons were carried out via a one-way analysis of variance followed by Tukey’s post hoc test. A P-value less than 0.05 was considered statistically significant. Data were expressed as means ± Standard Deviation (n = 5) [45].

## Results

### BUN assay

**Fig. 1:**
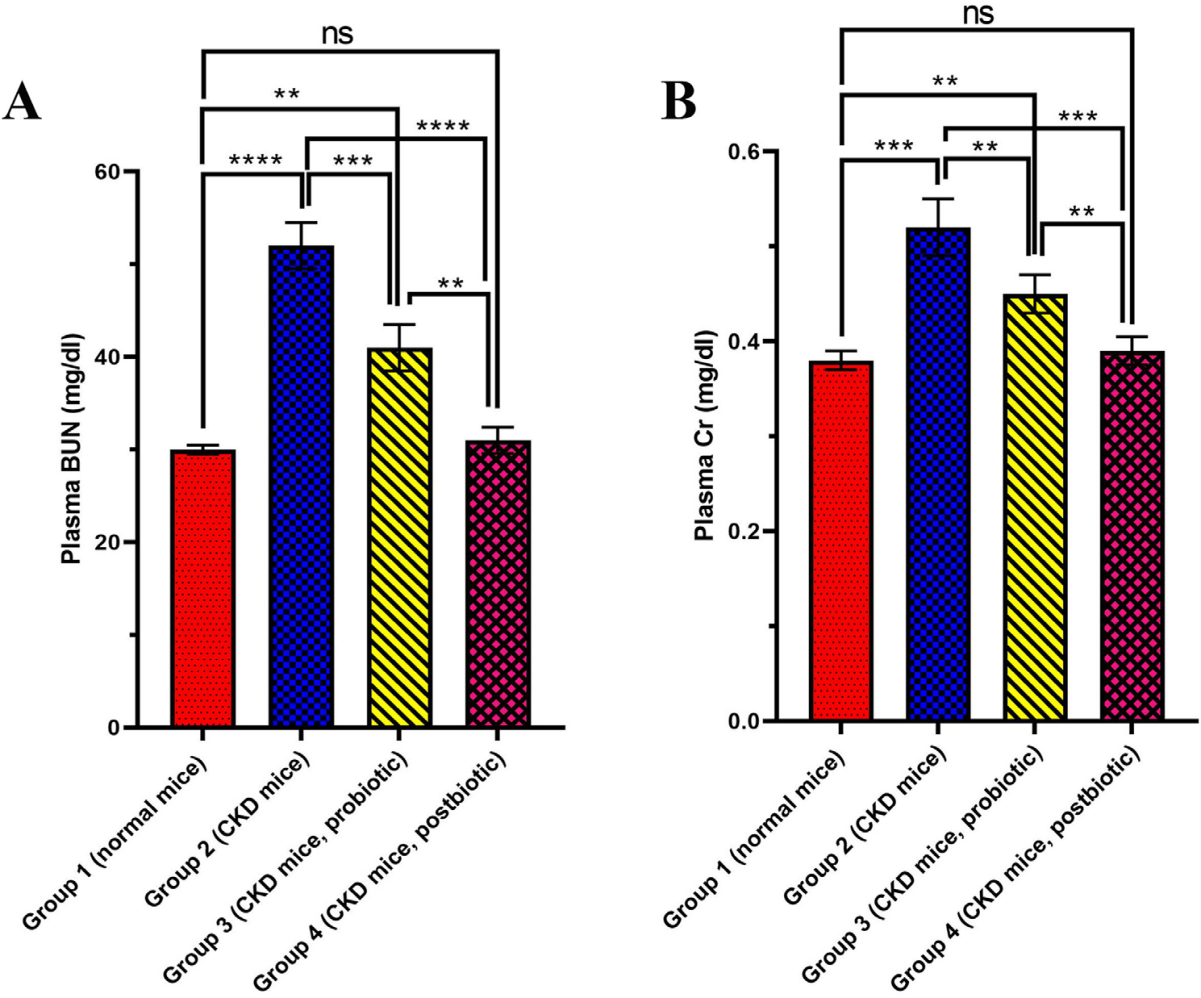
**(A)** Blood urea nitrogen (BUN) and **(B)** creatinine (Cr) levels, in different mice groups of Group 1 (normal mice, no treatment), Group 2 (Chronic kidney disease (CKD) mice, no treatment, Group 3 (CKD mice, *L. fermentum* probiotic treatment) and Group 4 (CKD mice, *L. fermentum* postbiotic treatment) at the end of Day 28. Data are expressed as Mean ± Standard deviation (SD) for five replicates. * (P < 0.05), ** (P < 0.01), *** (P < 0.001), **** (P < 0.0001), ***** (P < 0.00001) and ****** (P < 0.000001) and ns (not significant) are different levels of significance

Various CKD-induced mice groups were orally treated by *L. fermentum*, postbiotic or nothing, and then evaluated for the BUN level. As the results showed (Fig. 1A), cisplatin treatment (Group 2) significantly elevated the BUN level to 50.0 ± 3.0 mg/dl (P < 0.0001) compared to the control group (Group 1, 30.0 ± 1.3 mg/dl), which is healthy mice with normal renal function. Among all the groups, the value of BUN was the highest for the cisplatin-treated group that received no treatment (Group 2). Treatment of cisplatin-induced CKD groups with probiotic bacteria (Group 3) and the supernatant (postbiotic, Group 4) significantly decreased BUN level (P < 0.001 and P < 0.0001, respectively) in comparison to the cisplatin-treated group that received no treatment (Group 2). Among all the groups, the BUN level was the least (P < 0.0001) for the control group (Group 1, 30.0 ± 1.3 mg/dl) as well as the group that received the postbiotic (Group 4, 31.0 ± 1.4 mg/dl), the levels were not significantly different from each other (P > 0.05), followed by the group that received the probiotic (Group 3, 41.0 ± 2.5 mg/dl).

### Creatinine (Cr) assay

The effects of *L. fermentum* and postbiotic on Cr levels in cisplatin-induced CKD are demonstrated in Fig. 1B. Accordingly, cisplatin treatment (Group 2) led to a significant increase (P < 0.001) of Cr level to 0.52 ± 0.03 mg/dl compared to 0.38 ± 0.01 mg/dl for the control group (Group 1). Among all the groups, the Cr level was the maximum for the cisplatin-treated group without treatment (Group 2). It was shown that treatment of mice groups with probiotic bacteria (Group 3) and the supernatant (Group 4, postbiotic) significantly reduced the Cr level (P < 0.01 and P < 0.001, respectively) in comparison to the cisplatin-treated group, which received no treatment (Group 2). The Cr level was 0.45 ± 0.02 mg/dl for the group that received probiotics (Group 3). The Cr level was the minimum for the control group (Group 1, 0.38 ± 0.01 mg/dl, P > 0.001), which was as much as the group that received postbiotic (Group 4) (0.39 ± 0.01 mg/dl, P > 0.05).

### ROS formation

**Fig. 2:**
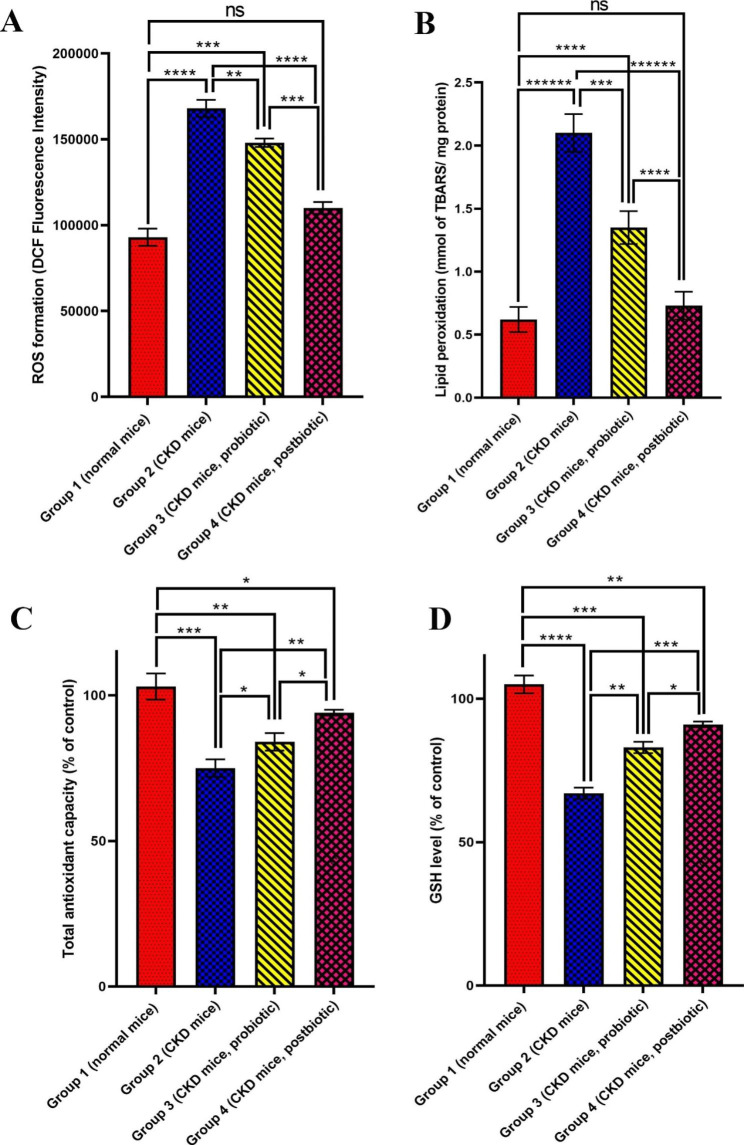
**(A)** Renal oxidative species (ROS) formation, **(B)** lipid peroxidation, **(C)** total antioxidant capacity, and **(D)** glutathione (GSH) level in kidney tissue in different mice groups of Group 1 (normal mice, no treatment), Group 2 (Chronic kidney disease (CKD) mice, no treatment, Group 3 (CKD mice, *L. fermentum* probiotic treatment) and Group 4 (CKD mice, *L.fermentum* postbiotic treatment) at the end of day 28. Data are expressed as Mean ± Standard deviation (SD) for five replicates. * (P < 0.05), ** (P < 0.01), *** (P < 0.001), **** (P < 0.0001), ***** (P < 0.00001) and ****** (P < 0.000001) and ns (not significant) are different levels of significance

As presented in Fig. 2A, the ROS increased after mice were treated with cisplatin alone (Group 2, P < 0.0001) in comparison to the control group. Probiotics (Group 3) and postbiotics (Group 4) significantly attenuated ROS formation (P < 0.01 and P < 0.0001, respectively) in comparison to the cisplatin-treated group without treatment (Group 2). The amount of ROS in kidney tissue in the control group (Group 1) is the lowest of all groups (P < 0.0001). Among the two groups of 3 and 4, the postbiotic (Group 4) displayed the most suppressive effect against ROS formation in cisplatin-induced CKD (P < 0.001).

### Tissue lipid peroxidation

The effects of probiotics and postbiotics on lipid peroxidation are shown in Fig. 2B. The antineoplastic agent, cisplatin, significantly augmented lipid peroxidation amount (Group 2, 2.10 ± 0.2 mmol TBARS/mg protein) compared to the control group (Group 1, 0.60 ± 0.07 mmol TBARS/mg protein, P < 0.000001). Treatment with the probiotic (Group 3) and postbiotic (Group 4) significantly declined lipid peroxidation compared to the cisplatin-treated mice group (Group 2) (P < 0.0001 and P < 0.000001, respectively). The result indicates that the postbiotic-treated mice group (Group 4) showed high efficacy in lipid peroxidation decrease (0.73 mmol ± 0.09 TBARS/mg protein, P < 0.00001), whose value was near the control group (Group 1) (P > 0.05).

### Total antioxidant capacity

Total antioxidant capacity (% of control) following various treatments of mice groups with *L. fermentum* probiotic and postbiotics is shown in Fig. 2C. It was revealed that total antioxidant capacity significantly decreased after administration of cisplatin (Group 2, 75% ± 3, P < 0.001) compared to the control group (Group 1, 102% ± 5). Supplementation with *L. fermentum* probiotic (Group 3) and postbiotic (Group 4) could improve the total antioxidant capacity. However, the postbiotic (Group 4) displayed the more ameliorative effect in comparison to the probiotic (Group 3) (P < 0.05).

### GSH level

Figure 2D presents the GSH level (% control) in different cisplatin-induced CKD mice groups treated with *L.fermentum* probiotic and postbiotic. The GSH level significantly decreased after cisplatin administration (Group 2, 67% ± 2, P < 0.0001) compared to the control group (Group 1, 104% ± 3). Supplementation with probiotics (Group 3) and postbiotics (Group 4) considerably enhanced the GSH levels in comparison to the cisplatin-treated group without treatment (P < 0.01 and P < 0.001). However, the postbiotic (Group 4) presented the better effect than the probiotic (Group 3)(P < 0.05).

### Histopathological study

**Fig. 3:**
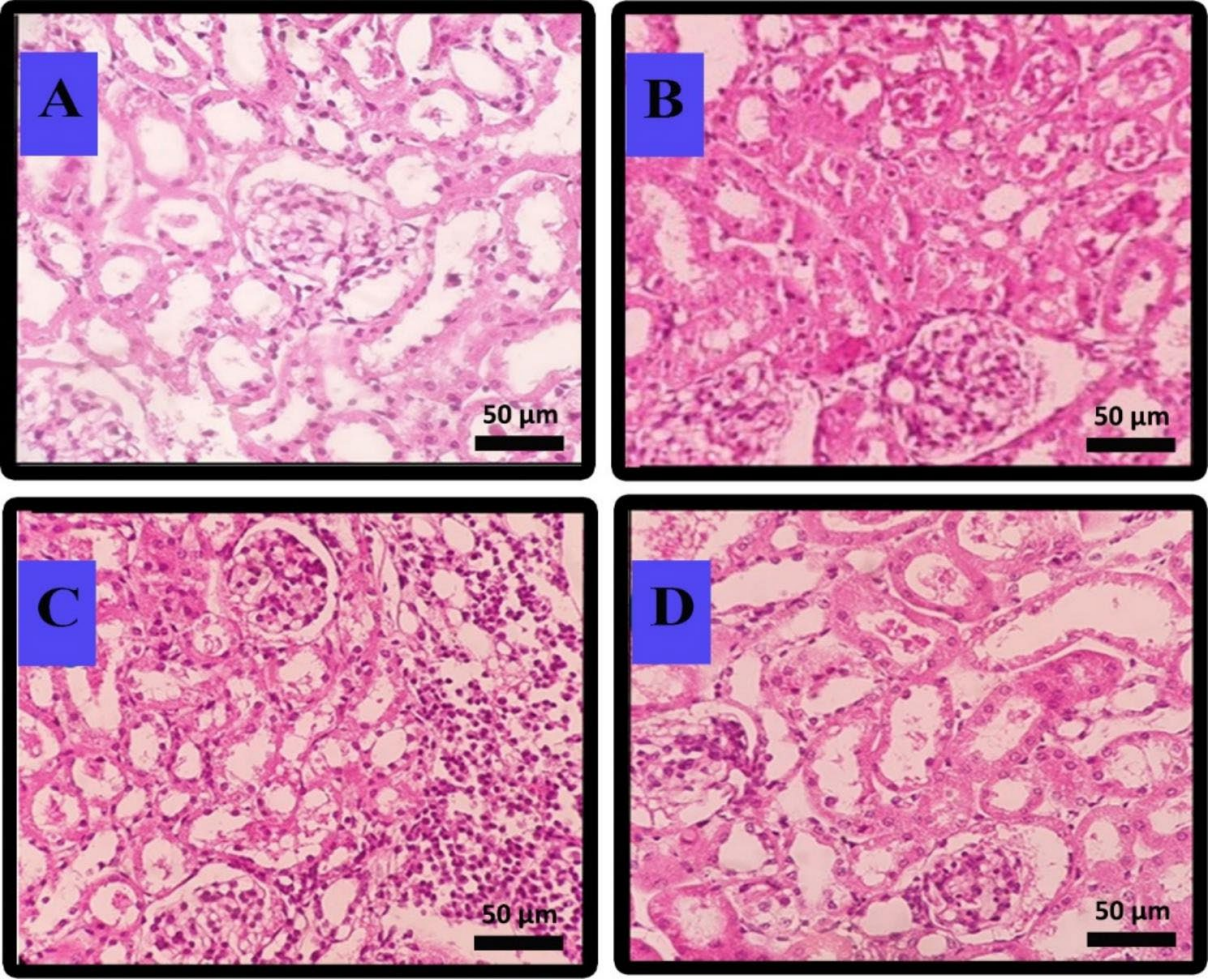
Histopathological assessment of kidney tissue in different mice groups at Day 28 through hematoxylin–eosin staining. **(A)** Normal mice group receiving no treatment (Group 1), **(B)** chronic kidney disease (CKD) induced mice group receiving no treatment (Group 2), **(C)** chronic kidney disease (CKD) induced mice group receiving *L. fermentum* probiotic bacteria (Group 3) and **(D)** chronic kidney disease (CKD) induced mice group receiving postbiotic (Group 4). Magnification, X 400

The damage percentage of kidney tissues and the histological sections of the kidneys related to different cisplatin-induced CKD mice following treatment with *L. fermentum* probiotics and postbiotics are shown in Table 2; Fig. 3. The results indicated that 19% of tubular and 10% of interstitial cells were damaged after cisplatin administration (Group 2). At the same time, no cytotoxic effects were detected in glomerular and vascular cells (Table 2; Fig. 3B). The tubular cell damage decreased after the administration of probiotic (Group 3, Table 2; Fig. 3C), and postbiotic (Group 4, Table 2; Fig. 3D) to 13% and 1%, respectively. In the case of interstitial cells, the cell injury was attenuated to 1% following postbiotic administration (Group 4).

**Table 2:** Kidney histopathological changes at the end of Day 28 in different mice groups

Changes	Group 1 Control mice	Group 2 Cisplatin-induced CKD group	Group 3 Cisplatin-induced CKD mice group receiving *L. fermentum* probiotic bacteria	Group 4 Cisplatin-induced CKD mice group receiving *L. fermentum* postbiotic
**Glomerular atrophy**	No noticeable change	No noticeable change	No noticeable change	No noticeable change
**Tubular atrophy**	No noticeable change	19%	13%	1%
**Interstitial nephritis**	No noticeablechange	10%	10%	1%
**Vascular changes**	No noticeable change	No noticeable change	No noticeable change	No noticeable change

## Discussion

CKD is a universal public health problem affecting millions, and its prevalence has recently increased. Discovering new therapeutic agents can be considered essential to slow the progression of CKD. Interestingly, there has been growing use of nutritional and natural remedies for managing various chronic diseases, which have shown promising effects [46–48]. Probiotics, particularly their bioactive metabolites, called bacterial supernatant postbiotics, have recently attracted considerable interest in various research areas due to their beneficial characteristics. The present study investigated the possible effects of L. fermentum probiotic and postbiotic on a cisplatin-induced CKD mice model through multiple assays.

Cisplatin was utilized to induce CKD in mice because of its ability to cause nephrotoxicity as one of its determining side effects [49]. This study compared the mice group receiving cisplatin (CKD-induced mice) without post-treatment with the standard mice group receiving no treatment in all assays (Figs. 1, 2 and 3). The results revealed that cisplatin administration could lead to a significant increase of BUN, Cr, ROS, and lipid peroxidation while a significant reduction of antioxidant capacity and GSH, which indicates the efficacy of the CKD induction cisplatin in the mice is observed.

CKD is usually linked with BUN or serum creatinine [50], and their elevations are considered nephrotoxicity indices [51]. The results of BUN (Fig. 1A) and Cr (Fig. 1B) assays suggest the high efficacy of the probiotic and the better efficacyof its metabolites (postbiotic) in reducing BUN and Cr levels towards the normal ones in CKD. Similar to our results, several in-vivo studies and clinical trials have shown the benefits of probiotic supplementation in reducing BUN and creatinine levels. An *in vivo* study reported that using *L. fermentum* decreased BUN and Cr levels significantly near normal conditions in lead-induced oxidative damage model in rats [52]. In another study, administering *Lactobacillus casei* Shirota to CKD-induced rats reduced BUN and Cr levels [53]. Another study observed that the BUN level was decreased in nephrectomized animals after being fed with a probiotic of *Lactobacilli*, *Bifidobacteria*, and *Streptococcus thermophilus* [54]. In addition, it was revealed that treatment with probiotic *Sporosarcina pasteurii* strain 6452 could significantly reduce the BUN level and increase the life spans of the nephrectomy rats [55]. Also, it was revealed that the BUN levels decreased and the life quality improved significantly in patients with CKD stages 3 and 4 after treatment with various probiotics such as *Lactobacillus acidophilus*, *Streptococcus thermophilus*, and *Bifidobacterium longum* [56] and also *Lactobacillus casei* Shirota [51].

The administration of the probiotic and postbiotic led to a significant reduction of the ROS level, indicating that these supplements can improve the disease conditions to normal. However, the postbiotic could result in the highest decrease of ROS to a level like the normal one. Regarding lipid peroxidation assay (Fig. 2B), treatment with probiotics and postbiotics demonstrated a significant reduction in lipid peroxidation, indicating their ability to decrease the CKD severity. The postbiotics were the most effective treatment among all tested groups. According to Fig. 2C, it was demonstrated that treatment with a probiotic and postbiotic could significantly increase the total antioxidant capacity, which was attenuated in cisplatin-induced CKD. The increase in TAC following treatment can be an indicator of CKD improvement.

Interestingly, the highest rise of antioxidant capacity was attributed to the postbiotic treatment. The obtained results agreed with several studies in which the antioxidant properties of probiotics have been confirmed and reported [57]. Also, as presented in Fig. 2D, the administration of probiotics and postbiotics significantly attenuated the GSH level decline in cisplatin-induced CKD. The maximum increase was related to the postbiotic, which suggests its highest efficacy in ameliorating CKD regarding GSH level. GSH could protect the cisplatin damage to the kidney through different mechanisms, such as improving the kidney’s function in the clearance of BUN and Cr and decreasing the renal production of malondialdehyde (MDA) as an index of lipid peroxidation [58]. *L. fermentum* can attenuate lipid peroxidation and oxidative damage through efficient scavenging of active free radicals of oxygen near cells and regulate the signal pathways associated with antioxidation in host cells, protecting the body from oxidative stress [52, 59]. It was shown that *L. fermentum* could decrease ROS levels while increase GSH in different body tissues such as serum, liver, and kidney, resulting in high antioxidant capacity [52]. In addition, it was demonstrated that *L. fermentum* could decrease inflammation.

Furthermore, *L. fermentum*  could activate the response of a signaling pathway named Keap1/Nrf2/ARE to secrete more antioxidant molecules. Besides, it can stimulate the expression of several genes to produce HO-1, NQO1, and γ-GCS, which have antioxidant capacity. HO-1 enhances the body’s ability to resist oxidative stress and cell damage. NQO1, a soluble flavone ubiquitous in almost all animal species, avoids the one-electron reduction of some toxic free radicals and preserves the reduced form of fat-soluble antioxidants to protect the body from oxidative stress. γ-GCS is an antioxidant factor of the Keap1/Nrf2/ARE signaling pathway. The rate-limiting enzyme in GSH biosynthesis scavenges many free radicals to decrease cell oxidative damage. Following the increase of these chemical productions, HO-1, NQO1, and γ-GCS, the oxidative stress response induced by a health problem would be reduced.

The histopathological results of kidney tissue showed that its use could lead to fewer glomeruli and tubular damage and less inflammation following the administration of *L. fermentum*. Interestingly, the liver and kidneys treated with *L. fermentum* were similar to the morphology obtained for the control group [52]. In the current study, probiotic therapy attenuated cisplatin-induced damage to the renal cells (19% in tubular and 10% in interstitial). However, according to results of different assays performed in the present study, the postbiotic treatment could show a complete recovery of the tubular and interstitial damages (approximately 1% of injuries remained). These findings are in agreement with the histopathological analysis of previous studies using probiotics in CKD, in which inflammation and damage of various parts of the kidney tissue decreased significantly [60–62]. The deterioration of kidney tissues revealed that the *L. fermentum* derivative’s administration attenuated CKD progression, and the postbiotic was much more effective. No detrimental effects of cisplatin were observed in glomeruli and vascular cells. These results demonstrate that the 4-week evaluation period in the present study needs to be revised, and the follow-up time should be prolonged to assess these factors.

The ameliorative effects of *L. fermentum* and particularly postbiotics against cisplatin-induced CKD in the current study may be related to various properties of the probiotics and postbiotics, including anti-inflammatory as well as antioxidant properties and their impact on increasing the antioxidant capacity and GSH levels [63]. One of the leading causes of chronic inflammation in CKD is dysbiosis. This situation is evident in the early stages of CKD, which creates a pro-inflammatory environment in the host. In this situation, bacteria with destructive enzymes such as urease, indole, and p-cresol increase and cause uremic toxins to accumulate in body fluids and help inflammation. Under conditions of microbial imbalance in the gut, epithelial tight junctions are damaged, the permeability of the gut barrier increases, and pathogenic bacterial products such as lipopolysaccharides leak into the circulation, leading to inflammation. Probiotics help reduce uremic toxins by replacing the composition of the gastrointestinal microbiota and positively competing with pathogens for nutrients and receptor binding sites.

Few studies investigate the efficacy of postbiotics in various health problems. Also, there have not been any studies on kidney diseases because it is recognized as a novel member of the -biotics family. However, some studies revealed that probiotic metabolites (and maybe postbiotics) directly attenuate the activation of pro-inflammatory nuclear factor (NF-κB) due to reduced lipo-polysaccharides (LPS). Also, they may modulate the gut microbiota and, potentially, the inflammatory state. In our study, probiotic and prebiotic inflammation status was independent of all these dietary factors. Recent research suggests that postbiotics plays a significant role in preserving and improving kidney health in preclinical studies. Lee et al. showed a beneficial effect of lacto-GABA-salt and postbiotics-GABA-salt (obtained from the fermentation of *Lactobacillus plantarum BJ21*) against cisplatin-induced renal histological changes [64]. Unfortunately, they do not provide details on the compounds obtained or the postbiotic composition. The reason behind the more efficacy of postbiotics than probiotics in the present study might be due to the concentration of these secreted bioactive substances in the postbiotic formulation. When postbiotics are used, responsible bioactive substances, such as functional proteins, extracellular polysaccharides, enzymes, cell wall fragments, and short-chain fatty acids, may exist in higher concentrations than the probiotics that have been utilized. Postbiotics present their therapeutic activities via different mechanisms, including modulation of the systemic and local immune response, helping the proper microbiome balance, and, therefore, modification of their metabolites. The postbiotics can also augment the epithelial barrier, modulate the immune system response, and modify metabolic activities and system signaling via the peripheral and central nervous systems [65].

In a review study, Favero et al. acknowledged that the therapeutic strategy of postbiotics was still defined as targeting downstream signaling pathways of the gastrointestinal microbiome. They proposed a roadmap for the clinical application of postbiotics for kidney disease [66]. It is worth noting that postbiotics have potent therapeutic effects on cisplatin-induced CKD caused by other factors. Further research is needed to verify the beneficial effects of certain ingredients of postbiotics on humans and other animals with kidney diseases to elucidate the detailed mechanisms of the renoprotective effects. To achieve the desired protective effect against nephrotoxicity, researchers should consider all aspects of the relevant mechanisms and take comprehensive measures or combinations of drugs. Furthermore, advancements of molecular biology technology has led to performing researches that rely on targeted therapy using postbiotics or derivatives highly selective for the kidney as carriers, chemically coupling these factors into biological treatments.

Postbiotics may have multiple active targets rather than only one unique target. Therefore, a postbiotic product may play various roles, exhibit wide use, and even have increased potential toxicity or side effects. Since some pathways of cisplatin-induced kidney injury are also involved in the antitumor effects of cisplatin, postbiotics may also affect cisplatin-mediated antitumor efficacy. In the future perspective of postbiotics, it is critical to identify the most effective probiotics in the development and progression of kidney health and define the mechanisms underlying their beneficial function, as not all bacterial components may be involved. For the comprehensive application, it is also necessary to overcome the limitations of postbiotic formulations, especially the problem of their instability due to time and temperature. Postbiotics should be designed and formulated to be more effective so that they do not undergo degradation and denaturation until the desired target in the body is reached. The production, route of administration, and definite characterization are the other problems of using postbiotics in kidney diseases.

The batch-to-batch variability of the manufactured postbiotics and scalability are other concerns that must be resolved. Moreover, more complex postbiotics consisting of diverse bacteria encoding beneficial metabolites in CKD conditions may be explored. Finally, randomized clinical trials in patients with kidney diseases should be designed. Short-term clinical trials should establish their safety in humans and examine biomarkers of their biological activity (e.g., oxaluria in patients with hyperoxaluria). Performing clinical trials focusing on broader efficacy endpoints may be more challenging in terms of methodology and financial resources, but they are required to gather the datasets necessary for approval by the medicine’s regulatory agencies. Finally, utilizing multiple dosing models (e.g., two doses of 15 mg/kg cisplatin, 2 weeks apart) may be more reflective of CKD caused by cisplatin in real preclinical setting than that we used in the current study (single dose of 10 mg/kg cisplatin).

## Conclusion

The efficacy of *L. fermentum* probiotic and postbiotic administration in a CKD mice model was evaluated through various biological assays as well as histopathological study. *L. fermentum* probiotic and postbiotic treatment of cisplatin-induced CKD mice resulted in protective effects by decreasing BUN, Cr, ROS formation, and lipid peroxidation levels while increasing TAC (total antioxidant capacity) and GSH levels, in which the postbiotic effects were higher. The histopathologic findings also confirmed the results of the biological assays. These results indicated that the tubular and interstitial cell damages decreased significantly following probiotic and postbiotic administration. It can be pointed out that treatment with probiotics and postbiotics could improve CKD in all the studied factors. However, the postbiotic-treated mice group (Group 4) had more significant improvement than the probiotic-treated mice group (Group 3).

## Data Availability

All data generated or analyzed during this study are included in this published article.
